# Fast Random Permutation Tests Enable Objective Evaluation of Methods for Single-Subject fMRI Analysis

**DOI:** 10.1155/2011/627947

**Published:** 2011-10-23

**Authors:** Anders Eklund, Mats Andersson, Hans Knutsson

**Affiliations:** ^1^Division of Medical Informatics, Department of Biomedical Engineering, Linköping University, Linköping, Sweden; ^2^Center for Medical Image Science and Visualization (CMIV), Linköping University, Linköping, Sweden

## Abstract

Parametric statistical methods, such as *Z-*, *t-*, and *F-*values, are traditionally employed in functional magnetic resonance imaging (fMRI) for identifying areas in the brain that are active with a certain degree of statistical significance. These parametric methods, however, have two major drawbacks. First, it is assumed that the observed data are Gaussian distributed and independent; assumptions that generally are not valid for fMRI data. Second, the statistical test distribution can be derived theoretically only for very simple linear detection statistics. With nonparametric statistical methods, the two limitations described above can be overcome. The major drawback of non-parametric methods is the computational burden with processing times ranging from hours to days, which so far have made them impractical for routine use in single-subject fMRI analysis. In this work, it is shown how the computational power of cost-efficient graphics processing units (GPUs) can be used to speed up random permutation tests. A test with 10000 permutations takes less than a minute, making statistical analysis of advanced detection methods in fMRI practically feasible. To exemplify the permutation-based approach, brain activity maps generated by the general linear model (GLM) and canonical correlation analysis (CCA) are compared at the same significance level.

## 1. Introduction

Functional magnetic resonance imaging (fMRI) is used in neuroscience and clinic for investigating brain activity patterns and for planning brain surgery. Activity is detected by fitting an activity model to each observed fMRI voxel time series and then testing whether the null hypothesis of no activity can be rejected or not based on the model parameters. Specifically, this test is performed by subjecting a test statistic calculated from the model parameters to a threshold. To control the randomness due to noise in this test procedure, it is desirable to find the statistical significance associated with the detection threshold, that is, how likely it is that a voxel is declared active by chance. When the statistical distribution of the data is known *and* when the probability (null)distribution of the test statistic can be derived, parametric statistics can be used to this end. This is for example the case for the commonly used general linear model (GLM), for which the well-known *t*-test and *F*-test can be derived when the input data are independently Gaussian distributed. However, when the data distribution is not known or the distribution of the test statistics cannot be derived, parametric statistical tests can only yield approximate thresholds or cannot be applied at all. This is generally the case in fMRI analysis as the noise in fMRI data is not Gaussian and independent [1–5]. Even though the noise can be made uncorrelated through a whitening procedure [6, 7], the noise structure must first be estimated using methods that themselves are susceptible to random errors. Accurately accounting for this variance in the test statistic distribution is difficult. Furthermore, more advanced detection approaches often adaptively utilize the spatial context of fMRI activation patterns to improve the detection, or they perform other operations that make the derivation of the test statistic distribution mathematically intractable [8–14]. Said otherwise, only for the very simplest test statistics, such as the GLM, can a parametric test distribution be derived theoretically. On top of the problems described above, the multiple testing problem [15] must be solved because one is generally interested to test whether there is any activity in the entire brain at all and not just if there is activity in a single voxel. This complicates the derivation of the test statistic distribution even further. To conclude, the parametric statistical approach is applicable only to a very limited set of tests and is subject to many sources of error.

In contrast to the parametric approach, a nonparametric approach does not assume the statistical properties of the input data to be known [16]. Furthermore, there is no need to derive the theoretical distribution of the test statistic, and even thresholds corrected for multiple testing are straightforwardly found. Nonparametric approaches have been studied extensively in functional neuroimaging [10, 17–28]. Semiparametric approaches have also been proposed [29]. In particular, so-called resampling or permutation methods have been studied, which randomly permute or reshuffle the original fMRI data to remove any activation signal but otherwise keep its statistical structure. Thus, thousands of simulated null data sets without activation can be generated and analysed to empirically simulate the null distribution of the test statistic. The major drawback of nonparametric statistical approaches for single-subject fMRI analysis is the computational complexity, requiring hours or days of processing time on regular computer hardware.

Graphics processing units (GPUs) have seen a tremendous development during the last decade and have been applied to a variety of fields to achieve significant speedups, compared to optimized CPU implementations. The main difference between the GPU and the CPU is that the GPU does all the calculations in parallel, while the CPU normally does them in serial. In the field of neuroimaging and neuroscience the use of the GPU is quite new. As single-subject fMRI analysis normally is done separately for each time series in the fMRI data, it suits perfectly for parallel implementations. In our recent work [30] we therefore describe how to preprocess (i.e., apply slice timing correction, motion correction, smoothing, and detrending) and how to statistically analyze the fMRI data on the GPU. The result is a significantly faster analysis than if the CPU is used. For a small fMRI dataset (80 volumes of the resolution 64 × 64 × 22 voxels) all the preprocessing is done in about 0.5 s and the statistical analysis is done under 0.5 ms. Recently, GPUs have also been used to speed up functional connectivity analysis of fMRI data [31, 32]. A final example is the work by Ferreira da Silva [33] that used the GPU to speed up the simulation of a Bayesian multilevel model for fMRI analysis. 

In this work, it is shown how nonparametric statistics can be made practical for single-subject fMRI analysis by using the parallel processing power of the GPU. The idea of using the GPU for random permutation tests is not new; it has recently been done in biostatistics [34, 35]. The GPU makes it possible to estimate the null distribution of a test statistic, corrected for multiple testing, in the order of minutes. This has significant implications on the way fMRI analysis can be carried out as it opens the possibility to routinely apply more powerful detection methods than the GLM. As an example, the results of the standard GLM detection is in this work compared with a restricted canonical correlation analysis (CCA) method [9] that adaptively incorporates spatial context in the detection. The short processing time also facilitates deeper investigations into the influence of various noise and detrending models on the statistical significance, as well as validation of approximate parametric approaches such as Bonferroni and random field theory (RFT) [36–39].

## 2. Methods

### 2.1. Basics of Random Permutation Tests

**Figure 1:**
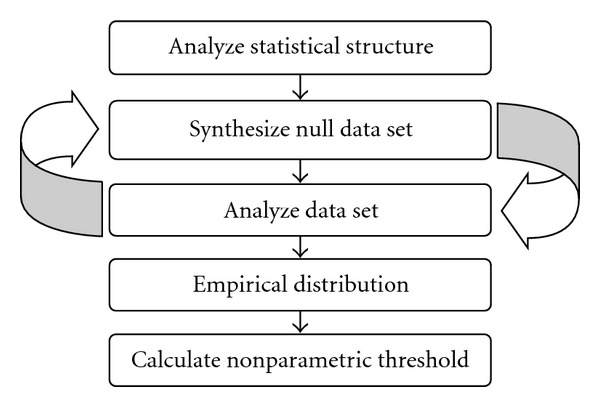
Flowchart containing the main building blocks for nonparametric analysis of single-subject fMRI data.

One subset of nonparametric tests is permutation tests where the statistical analysis is done for all the possible permutations of the data. Complete permutation tests are; however, not feasible if the number of possible permutations is very large. For a time series with 80 samples, there exists 7.16 · 10^118^ possible permutations. It is therefore common to instead do *random* permutation tests [40], also called Monte Carlo permutation tests, where the statistical analysis is made for a sufficiently large number of random permutations, for example 10 000, of the data. The main idea is to estimate the null distribution of the test statistics, by generating and analysing surrogate data that is similar to the original data. The surrogate data is generated by permuting, or reshuffling, the data between the different groups to be compared. The main idea of the nonparametric approach is given in Figure 1.

### 2.2. The Problem of Multiple Testing

By applying a threshold to the activity map, each voxel can be classified as active or inactive. The threshold is normally selected as a level of significance, one may for example want that only voxels that with at least 95% significance are to be considered as active. If a statistical test is repeated and a family-wise error rate *α* is desired, the error rate for each test must be smaller than *α*. This is known as the problem of multiple testing. If Bonferroni correction is used, the error rate for each comparison becomes *α*/*N*_*v*_, where *N*_*v*_ is the number of tests (voxels). This is a correct solution if the tests are independent. In fMRI it is common to perform the statistical analysis for more than 20 000 brain voxels; if a threshold of *P* = 0.05 is used to consider the voxel as active, the *P* value becomes 0.05/20000 with Bonferroni correction. The assumptions that are made about the behaviour of the tail of the distribution are thereby critical.

There are three problems with Bonferroni correction in fMRI. First, the test statistics is assumed to, under the null hypothesis, follow a certain distribution, such as Student's *t*-distribution. Second, the smoothness of the data is not taken into account as the Bonferroni threshold only considers the number of tests. The smoothing increases the spatial correlation of the data and thereby reduces the effective number of independent tests. Third, it is assumed that all the voxels have the same null distribution. To avoid Bonferroni correction, another approach based on Gaussian random field theory [36, 38, 39] has been developed and is used in the statistical parametric mapping (SPM) software (http://www.fil.ion.ucl.ac.uk/spm/). While this approach takes the smoothness of the data into account, several assumptions are necessary for the theory to be valid and it is still assumed that all the voxels have the same null distribution.

The nonparametric approach can be used to solve the problem of multiple testing as well. This is done by estimating the null distribution of the *maximum* test statistic [19, 21, 24, 39] by only saving the maximum test value from each permutation, to get a *corrected* threshold. This means that about 10 000 permutations have to be used [19, 21], while as little as 10 permutations can be enough if an *uncorrected* threshold is sufficient [18, 20, 22].

### 2.3. Preprocessing of fMRI Time Series

As fMRI time series are temporally correlated [6, 36, 41], the time series have to be preprocessed before they are permuted. Otherwise the exchangeability criterion is not satisfied and the temporal structure is destroyed. Most of the temporal correlations originate from different kinds of trends. In this work these trends are removed by a cubic detrending, such that the mean and any polynomial trend up to the third order is removed, but more advanced detrending is possible [42].

Several approaches have been proposed for the random resampling, the most common being whitening transforms [6, 18, 19], wavelet transforms [22, 26], and Fourier transforms [43]. A comparison of these approaches [27] indicates that whitening performs best, at least for fMRI data that is collected during block-based stimuli paradigms. The whitening transform is done by estimating an autoregressive (AR) model for each time series. This can, for example, be done by solving the equation system that is given by the Yule-Walker equations.

To accurately estimate AR parameters from a small number of time points (80 in our case) is quite difficult. To improve the estimates a spatial Gaussian lowpass filter (8 mm FWHM) is therefore applied to the estimated AR parameters [7]. In statistics this technique is normally known as variance pooling. The optimal amount of smoothing was found by testing the AR estimation procedure on temporally correlated Gaussian noise where the spatial patterns of the AR parameters were known. Our amount of smoothing (8 mm) is less than the first application of smoothing of the parameters (15 mm) [7] but close to the optimal amount of smoothing (6.5–7.5 mm) found by further investigation [44]. It has also been reported that the AR estimates are better without the spatial smoothing [45].

Normalized convolution [46] is used to prevent that the smoothing includes AR parameters from outside the brain. With normalized convolution it is possible to use a certainty value for each sample in the convolution. The certainty weighted filter response cwr is calculated as

(1) $$\text{cwr}=\frac{\left(c\cdot s\right)*f}{c*f},$$

where *c* is the certainty, *s* is the signal, *f* is the filter, · denotes point-wise multiplication, and ∗ denotes convolution. In our case the certainty is set to 1 for the brain voxels and 0 otherwise. Without the normalized convolution the estimated AR parameters at the edge of the brain are too low, as the AR parameters outside the brain are very close to 0. To further improve the estimates, the whitening procedure is iterated 3 times and the AR estimates are accumulated [7, 22] (a higher number of iterations seem to impair the estimates).

To investigate if the time series really are white noise after the whitening, several tests can be applied. One example is the Durbin-Watson test that previously has been used to test if the residuals from the GLM contain autocorrelation [2]. The problem with this test is, however, that it only tests if there is an AR(1) correlation or not, it cannot handle higher-order correlations. A more general test is the Box-Pierce test that tests if at least one of the autocorrelations up to a defined time lag *h* is significantly different from zero. The Box-Pierce test has also been used for testing whiteness of fMRI data [22]. The Ljung-Box test [47] has been proven to be better than the Box-Pierce test for small sample sizes and is therefore used in our case. The test statistic *Q* is calculated as

(2) $$Q=N_{t}\left(N_{t}+2\right)\overset{h}{\underset{k=1}{\sum }}\frac{{\left(r_{Y}\left(k\right)\right)}^{2}}{N_{t}-k},$$

where *N*_*t*_ is the number of time samples, *r*_*Y*_(*k*) is the sample autocorrelation at time lag *k*, and *h* is the number of time lags being tested. The test statistic is asymptotically chi-square distributed with *h* − *p* degrees of freedom, where *p* is the order of the AR model used for the whitening, when *N*_*t*_ grows towards infinity. Since our whitening is done with the smoothed AR parameters, the Ljung-Box test is applied to smoothed auto correlations.

Since the spatial correlation should be maintained, but not the temporal, the same permutation is applied to all the time series [19, 43]. When the time series have been permuted, an inverse whitening transform is applied by simulating an AR model, using the permuted whitened time series as innovations.

### 2.4. Statistical Analysis, GLM and t-Test

The general linear model (GLM) is the most used approach for statistical analysis of fMRI data [37]. For each voxel time series, a linear model is fitted according to

(3) Y=Xβ+ϵ,

where **Y** is the time series, **X** the regressors, **β** the parameters to estimate, and **ϵ** the errors. The regressors were created by convolving the stimulus paradigm with the hemodynamic response function (HRF) (difference of gammas) and its temporal derivative [6]. The two regressors were mean corrected, Euclidean normalized, and orthogonalized. No other regressors were used in the design matrix. The regression weights are estimated with ordinary least squares

(4) $$\hat{\beta }={\left(X^{T}X\right)}^{-1}X^{T}Y,$$

and the *t*-test value is then calculated as

(5) $$t=\frac{c^{T}\hat{\beta }}{\sqrt{\text{var}\left(\hat{\epsilon }\right)c^{T}{\left(X^{T}X\right)}^{-1}c}},$$

where **c** is the contrast vector.

Prior to the GLM the time series were whitened by using the same AR(1) model for all the voxels [6, 18]. No additional high-pass or low-pass filtering was used. The whitening step prior to the GLM is not necessary for the permutation-based analysis. The purpose of the whitening is to make sure that the errors are temporally uncorrelated, otherwise the assumptions that are necessary for the GLM to generate a true *t*-value are violated. Without the whitening a true *t*-value is not obtained, but a pseudo *t*-value. This is not a problem for the permutation-based analysis as the null distribution of the test statistics is estimated. If the thresholds from random field theory and a random permutation test are to be compared, the whitening has to be done in each permutation.

### 2.5. Statistical Analysis, CCA

One statistical approach for fMRI analysis that provides more adaptivity to the data is canonical correlation analysis (CCA) [48]. While the GLM works with *one* multidimensional variable (e.g., temporal basis functions, [37]), CCA works with *two* multidimensional variables (e.g., temporal and spatial basis functions, [9]). Ordinary correlation between two one-dimensional variables *x* and *y* with zero mean can be written as

(6) $$\rho =\text{Corr}\left(x,y\right)=\frac{E\left[xy\right]}{\sqrt{\;E\left[x^{2}\right]\;\text{E}\left[y^{2}\right]}}.$$

This expression can easily be extended to multidimensional variables. The GLM calculates the correlation between one multidimensional variable **x** and one one-dimensional variable *y* according to

(7) $$\rho =\text{Corr}\left(\beta^{T}x,y\right),$$

where **β** is the weight vector that determines the linear combination of **x**. Canonical correlation analysis is a further generalization of the GLM, such that both the variables are multidimensional. The canonical correlation is defined as

(8) $$\begin{array}{c} \rho =\text{Corr}\left(\beta^{\text{T}}x,\gamma^{\text{T}}y\right)=\frac{\beta^{T}C_{xy}\gamma }{\sqrt{\beta^{T}C_{xx}\;\beta \;\gamma^{T}C_{yy}\gamma }}, \end{array}$$

where **C**_**xy**_ is the covariance matrix between **x** and **y**, **C**_**xx**_ is the covariance matrix for **x**, and **C**_**yy**_ is the covariance matrix for **y**. The temporal and spatial weight vectors, **β** and **γ**, that give the highest correlation are calculated as the eigenvectors of two eigenvalue problems. The canonical correlation is the square root of the corresponding eigenvalue. 

**Figure 2:**
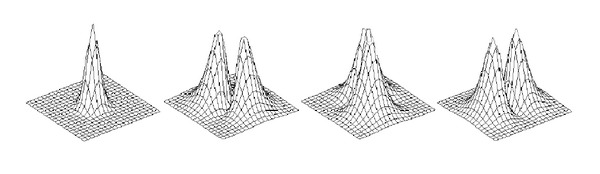
The four smoothing filters that are used for 2D CCA, one small isotropic separable filter and three anisotropic nonseparable filters. For visualization purposes, these filters are interpolated to a subpixel grid.

**Figure 3:**
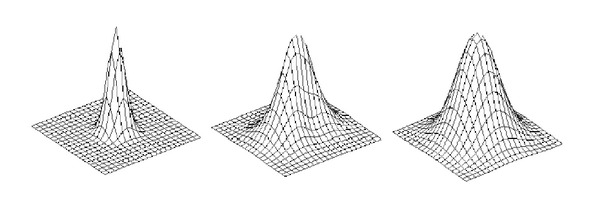
**Left**: A small isotropic lowpass filter can be used by CCA by setting the weights of all the other filters to zero. **Middle**: anisotropic lowpass filters with arbitrary orientation can be created by CCA by first combining the anisotropic filters and then adding the small lowpass filter. **Right**: by using the same weight for all the filters, a large isotropic lowpass filter can be obtained.

The temporal basis functions for CCA are the same as for the GLM. The spatial basis functions can, for example, be neighbouring pixels [8, 10, 49] or a number of anisotropic filters [9] that linearly can be combined to a lowpass filter with arbitrary orientation, to prevent unnecessary smoothing. In contrast to the GLM, an adaptive anisotropic smoothing is obtained, instead of a fix isotropic smoothing. The four smoothing filters that are used for our implementation of 2D CCA are given in Figure 2. Three filters that can be constructed as a linear combination of the four filters are given in Figure 3.

Compared to other approaches that adaptively include spatial information [11–14], the advantage with CCA is that there exists an analytical solution that gives the best weight vectors, while the other approaches have to search for the best combination.

One disadvantage with CCA is that it is difficult to calculate the threshold for a certain significance level, as the distribution of the canonical correlation coefficients is rather complicated. If **x** and **y** are Gaussian distributed and independent, the joint probability distribution for *all* the sample canonical correlation coefficients is given by [50]

(9) $$f=\overset{m}{\underset{i=1}{\prod }}\left({\left(r_{i}^{2}\right)}^{(n-m-1)/2}{\left(1-r_{i}^{2}\right)}^{(N-n-m-1)/2}\overset{m}{\underset{j=i+1}{\prod }}\left(r_{i}^{2}-r_{j}^{2}\right)\right),$$

where *N* is the number of (time) samples, *n* and *m* are the dimensions of the multidimensional variables **x** and **y**, and *r*_*i*_ are the canonical correlation coefficients.

Another problem is that restricted CCA (RCCA) [51] normally is used instead of ordinary CCA, in order to guarantee that the resulting combinations of the temporal and spatial basis functions are plausible. To our knowledge there is no theoretical distribution for restricted canonical correlation coefficients. The only solution to get a significance threshold for RCCA is thus to use nonparametric approaches.

As a 2D version of CCA already had been implemented [30], it was easy to extend the random permutation tests to include CCA as well. The problem with the original 3D CCA approach [9] is that it uses a total of seven 3D filters, and thereby a 7 × 7 matrix must be inverted for each time series. Our GPU implementation, however, only supports inverting 4 × 4 matrices, and thereby a maximum of 4 filters. Another approach [52] that uses two 3D filters, one isotropic Gaussian kernel and its derivative (with respect to the width parameter sigma), was therefore used. This makes it possible for CCA to create filters with different sizes, such that the amount of smoothing varies between the voxels. All the resulting filters are, however, isotropic, which makes this version of 3D CCA less adaptive.

### 2.6. Spatial Smoothing

The smoothing of the fMRI volumes has to be applied in each permutation. If the data is smoothed prior to the whitening transform, the estimated AR parameters will change with the amount of smoothing applied since the temporal correlations are altered by the smoothing. For our implementation of 2D CCA, 4 different smoothing filters are applied. If the smoothing is done prior to the permutations, 4 time series have to be permuted for each voxel and these time series will have different AR parameters. The smoothing will also change the null distribution of each voxel. This is incorrect as the surrogate null data that is created always should have the same properties, regardless of the amount of smoothing that is used for the analysis. If the data is smoothed after the whitening transform, but before the permutation and the inverse whitening transform, the time series that are given by simulating the AR model are incorrect since the properties of the noise are altered. The only solution to this is to apply the smoothing *after* the permutation and the inverse whitening transform, that is, in each permutation. This is also more natural in the sense that the surrogate data first is created and then analysed.

Similarly, if the activity map is calculated as how important each voxel is for a classifier [11–14], the classifier has to be trained in each permutation in order to estimate the null distribution.

### 2.7. The Complete Algorithm

The complete algorithm can be summarized as follows. The reason why the detrending is done separately, compared to having the detrending basis functions in the design matrix, is that the detrending has to be done separately for the CCA approach.

The whitening in each permutation is only performed to be able to compare the corrected *t*-thresholds from the random permutation test to the thresholds from Bonferroni correction and random field theory.

(1) Preprocess the fMRI data, that is, apply slice timing correction, motion correction, smoothing, and cubic detrending. To save time, the statistical analysis is only performed for the brain voxels. A simple thresholding technique is used for the segmentation.

(2) Whiten the detrended time series (GLM only).
(3) Apply the statistical analysis to the preprocessed fMRI data and save the test values. These are the original test values *t*_voxel_.

(4) Apply cubic detrending to the motion compensated time series.

(5) Remove the best linear fit between the detrended time series and the temporal basis functions in the design matrix, by ordinary least squares, to create residual data (as the *null* distribution is to be estimated). Estimate AR parameters from the residual time series. Apply a spatial smoothing to improve the estimates of the AR parameters. Apply whitening with the smoothed AR parameters. Repeat the whitening procedure 3 times.
(6) For each permutation,
  - apply a random permutation to the whitened time series,
  - generate new fMRI time series by an inverse whitening transform, that is, by simulating an AR model in each voxel with the permuted whitened time series as innovations,
  - smooth all the volumes that were generated by the inverse whitening transform,
  - apply cubic detrending to the smoothed time series,
  - whiten the detrended time series (GLM only),
  - apply the statistical analysis,
  - find the maximum test value and save it.

(7) Sort the maximum test values.

(8) The threshold for a desired corrected *P* value is given by extracting the corresponding test value from the sorted maximum test values. If 10 000 permutations are used, the threshold for corrected *P* = 0.05 is given by the sorted maximum test value at location 9500.

(9) The corrected *P* value at each voxel is calculated as the number of maximum test values, *t*max_*i*_, that are greater than or equal to the original test value in the voxel, *t*_voxel_, divided by the number of permutations *N*_*p*_

(10) $$p_{\text{voxel}}^{c}=\frac{\sum_{i=1}^{N_{p}}\left(t\text{ma}{\text{x}}_{i}\geq t_{\text{voxel}}\right)}{N_{p}}.$$

**Figure 4:**
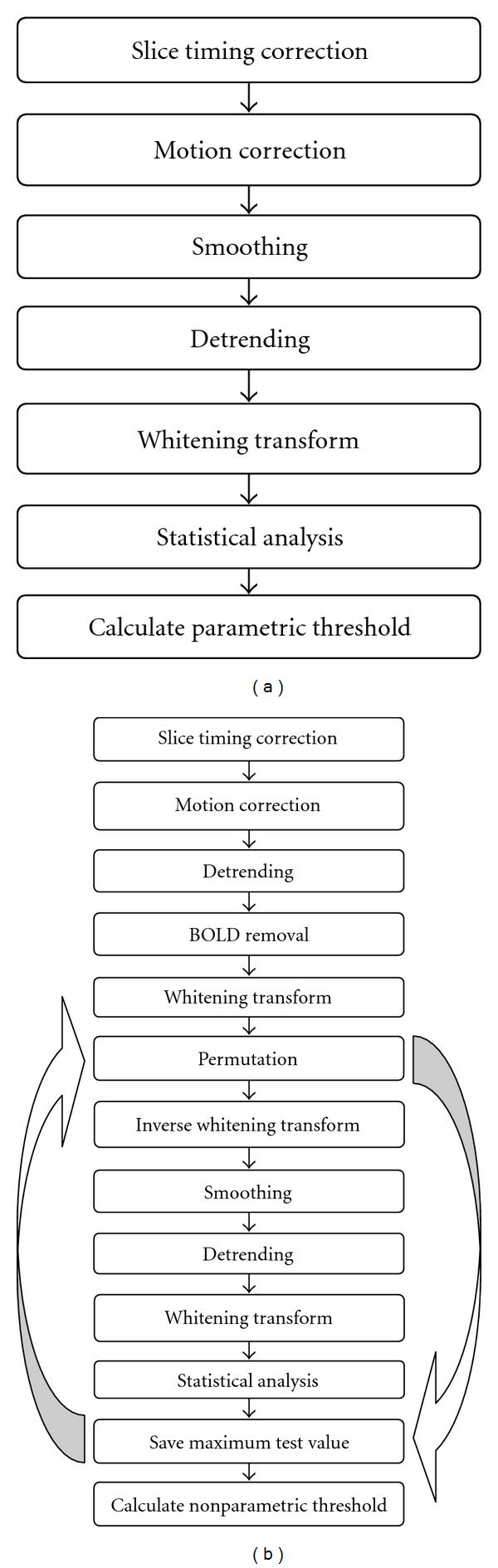
(a) Flowchart for conventional parametric analysis of fMRI data. (b) Flowchart for nonparametric analysis of fMRI data. In each permutation a new null dataset is generated and analysed.

A comparison of the flowcharts for a parametric analysis and a nonparametric analysis is given in Figure 4.

### 2.8. The Number of Permutations

The number of permutations that are required depends on the desired *P* value and the accuracy that is required. The standard deviation of the desired (one sided) *P* value is approximately $\sqrt{p(1-p)/N_{p}}$, where *N*_*p*_ is the number of permutations [53]. Some examples of desired *P* value, number of permutations, and relative standard deviation are given in Table 1.

## 3. GPU Implementation

The random permutation test was implemented with the CUDA (Compute Unified Device Architecture) programming language by Nvidia [54], which is explained by Kirk and Hwu [55]. In this section we will shortly describe how to implement the whitening transform and the random permutation test on the GPU. The interested reader is referred to our recent work [30] for more details and how to implement the other processing steps. The main principle of our GPU implementation is that each GPU thread works on a separate voxel time series.

Our CUDA implementation was compared with a standard *C* implementation and an OpenMP-based implementation. The Open MP (Open Multi-Processing) library lets the user take advantage of all the CPU cores in an easy way. All the implementations have been done in Matlab (Mathworks, Natick, Mass), using the mex interface where *C* and CUDA code can be used together with Matlab. For all implementations, 32 bit floats were used. The used graphics cards were three Nvidia GTX 480, each equipped with 480 processor cores and 1.5 GB of memory, giving a total of 1440 processor cores that run at 1.4 GHz. The used CPU was an Intel Xeon 2.4 GHz with 12 MB of L3 cache and 4 processor cores, and 12 GB of memory was used. The operating system used was Linux Fedora 12 64-bit. The total price of the computer was about 4000 $, a fraction of the price for a PC cluster with equivalent computational performance.

### 3.1. Whitening and the Random Permutations

Before the data is permuted an AR model is first estimated for each time series, as previously described. To solve the equation system that is given by the Yule-Walker equations requires a matrix inverse of the size *p* × *p* where *p* is the order of the AR model. To actually do the matrix inverse in each thread on the GPU is not a problem, even for matrices larger than 4 × 4, but to do it for a 7 × 7 matrix requires a lot of float registers and the Nvidia GTX 480 can only use 64 float registers per thread. The CUDA compiler will put the rest of the float variables, that do not fit into the registers, in the local memory which is extremely slow. Due to this it is hard to achieve good performance for matrices that are larger than 4 × 4. This is also the reason why the original 3D CCA approach, that uses seven 3D filters, cannot be used. Other than this the estimation of the AR parameters suits the GPU well, as the parameters are estimated in exactly the same way for each voxel time series. When the AR parameters have been estimated, they are spatially smoothed in order to improve the estimates. For this purpose a separable 3D convolver, created for 3D CCA and 3D GLM, is used.

For the estimation of AR parameters, the whitening transform, and the inverse whitening transform the shared memory is used to store the *p* last time points and each GPU thread loops along the time dimension for one voxel.

The permutation step is done by using randomized indices. A permutation matrix of size *N*_*p*_ × *N*_*t*_ is first generated in Matlab, by using the function *randperm*, and is then copied to the GPU. For each permutation one row of the permutation matrix is copied to the constant memory and is used to read the data in the randomized order. It might seem difficult to achieve coalesced reads when the time samples are to be read in a randomized order, in our case this is however not a problem. The fMRI data is stored as (*x*, *y*, *z*, *t*) (i.e., *x* first, then *y* and so on) and the permutation is only done along the time dimension, and not along the spatial dimensions. Due to this fact it is always possible to read 32 values at the time along *x*, regardless of the current time point. The code in Algorithm 1 generates a new time series for one voxel, by simulating an AR(4) model using the permuted whitened time series as innovations:

c_Permutation_Vector

is the index vector that contains the random time indices. The inverse whitening transform and the permutation step are thus performed at the same time. The help functions

Get_3D_Index, Get_4D_Index

calculate the linear index for the 3D and the 4D case. 

For this kernel, and for most of the other kernels, each thread block contains a total of 512 threads (32 along *x*, 16 along *y*, and 1 along *z*) and uses 16 KB of shared memory (one 16 × 8 × 32 float array) which makes it possible to run three thread blocks in parallel on each multiprocessor. This results in 1536 active threads per multiprocessor and thereby a total of 23 040 active threads on the GPU, which is the maximum for the Nvidia GTX 480.

To find the maximum test value in each permutation, one fMRI slice (64 × 64 pixels) is first loaded into shared memory. The maximum value of this slice is then found by comparing two values at the time. The number of values is thus first reduced from 4096 to 2048, then to 1024 and after 12 reductions to the maximum test value. The maximum values of the 22 slices are then compared. After each permutation the maximum test value is copied to host memory.

In order to calculate the *P* value for each voxel, the maximum test values are first copied from host memory to constant memory. Each GPU thread then loops over all the maximum test values and calculates how many of the test values are bigger than or equal to the test value for one voxel.

### 3.2. Multi-GPU

As our computer contains three graphic cards, a multi-GPU implementation of the analysis was also made, such that each GPU does one-third of the permutations. Each GPU first preprocesses the fMRI data, GPU 1 uses the first part of the permutation matrix, GPU 2 uses the middle part, and GPU 3 uses the last part. The processing time thus scales linearly with the number of GPUs. A short demo of the multi-GPU implementation can be found at http://www.youtube.com/watch?v=wxMqZw0jcOk.

## 4. Results

In this section we will present the processing times for the different implementations, compare activity maps from GLM and CCA at the same significance level, and compare estimated thresholds from Bonferroni correction, Gaussian random field theory, and random permutation tests.

### 4.1. Data

Four single-subject datasets have been used to test our algorithms; the test subject was a 50-year-old healthy male. The data was collected with a 1.5 T Philips Achieva MR scanner. The following settings were used: repetition time 2 s, echo time 40 ms, flip angle 90 degrees, and isotropic voxel size 3.75 mm. A field of view of 240 mm thereby resulted in slices with 64 × 64 pixels, and a total of 22 slices were collected every other second. The experiments were 160 s long, resulting in 80 volumes to be processed. The datasets contain about 20 000 within-brain voxels.

#### 4.1.1. Motor Activity

For the *Motor 1* dataset the subject periodically activated the left hand (20 s activity, 20 s rest), and for the *Motor 2* dataset the subject periodically activated the right hand.

#### 4.1.2. Language Activity

For the *Language* dataset the subject periodically performed a reading task (20 s activity, 20 s rest). The task was to read sentences and determine if they were reasonable or not.

#### 4.1.3. Null

For the *null* dataset the subject simply rested during the whole experiment.

### 4.2. Processing Times

The processing times for the random permutation tests, for the different implementations, are given in Tables 2 and 3. The reason why the processing time does not scale linearly with the number of permutations is that it takes some time to copy the data to and from the GPU. Before the permutations are started, the fMRI data is preprocessed on the GPU and this takes about 0.5 s. The processing times for the different processing steps can be found in our recent work [30].

The reason why the processing time for CCA is much longer than for the GLM for the CPU implementations is that the 2D version of CCA uses one separable filter and three nonseparable filters for the smoothing while the GLM uses one separable filter. For the GPU implementation the 2D smoothing can be done extremely fast by using the shared memory. 

### 4.3. Verifying the Whitening Procedure

**Figure 5:**
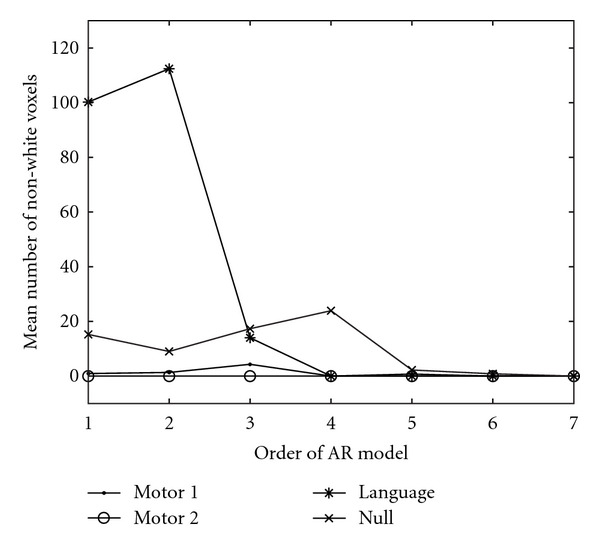
Mean number of voxels classified as nonwhite by the Ljung-Box test (1–10 time lags were tested and the mean number of nonwhite voxels for the 10 tests was saved). Prior to the Ljung-Box test the estimated auto correlations were spatially smoothed. The number of nonwhite voxels for Gaussian white noise is always zero.

**Figure 6:**
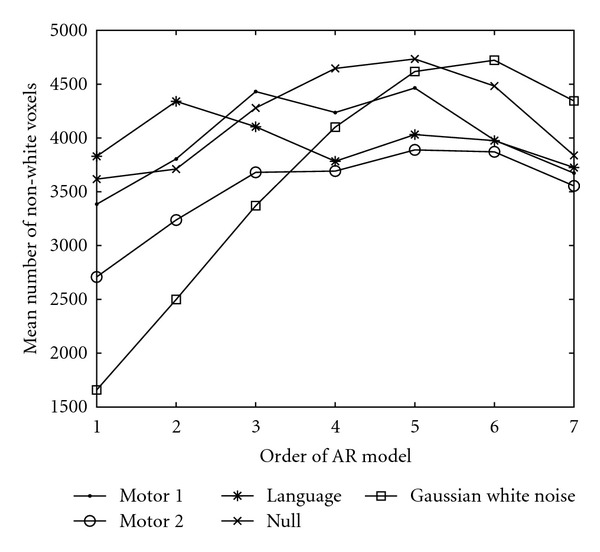
Mean number of voxels classified as nonwhite by the Ljung-Box test (1–10 time lags were tested and the mean number of nonwhite voxels for the 10 tests was saved). No spatial smoothing was applied to the estimated auto correlations prior to the Ljung-Box test. The number of nonwhite voxels for Gaussian white noise is included as reference (no whitening was applied to the noise).

To verify that the whitening procedure prior to the permutations works correctly, the Ljung-Box test was applied to each residual time series. The Ljung-Box test was applied to the four datasets after detrending, BOLD removal, and whitening with AR models of different order. The test was applied for 1–10 time lags (i.e., 10 tests), and the mean number of nonwhite voxels was saved. A voxel-wise threshold of *P* = 0.05 was used, that is, *χ*_0.95,*h*−*p*_^2^ where *h* is the number of time lags tested and *p* is the order of the AR model used. This means that the test only can be applied to certain time lags, since the degrees of freedom otherwise become zero or negative. The results with spatial smoothing of the auto correlations are given in Figure 5 and the results without spatial smoothing are given in Figure 6. The results for Gaussian white noise are included as reference when no smoothing is applied to the auto correlations. With the spatial smoothing, the number of voxels classified as nonwhite for the Gaussian noise is always zero.

If no smoothing is applied to the estimated auto correlations prior to the Ljung-Box test, the test statistic cannot be trusted as the standard deviation of the estimated auto correlations is too high. The reason why the number of nonwhite voxels increases, when no smoothing is applied to the auto correlations and when the degree of the AR model increases, is that the critical threshold of the Ljung-Box test decreases as the order of the AR model increases.

From the results in Figures 5 and 6 we draw the conclusion that cubic detrending and an individual AR(4) whitening is necessary to whiten the *Motor 1*, *Motor 2,* and *Language* datasets while an individual AR(5) or AR(6) whitening is necessary for the *Null* dataset. Long-term autocorrelations have previously been reported for resting state fMRI data.

For all the datasets an individual AR(4) whitening was therefore used prior to the permutations and in each permutation to generate new null data. For the *null* dataset a higher-order AR model is necessary, but to estimate an AR(5) model requires matrix inverses of 5 × 5 matrices for each voxel time series, which our GPU implementation does not support. Therefore, the voxels in the *null* dataset that were considered as nonwhite after the AR(4) whitening were instead removed from the random permutation test. 

### 4.4. Verifying the Random Permutation Test

**Figure 7:**
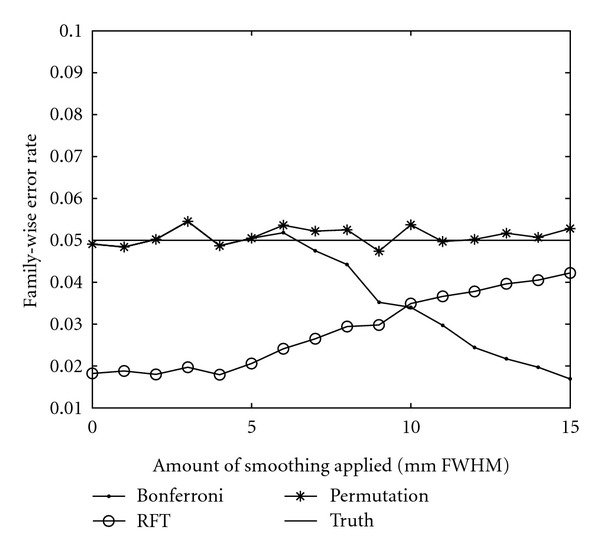
A comparison of family-wise error rates for Gaussian white noise for three different approaches to calculate an activity threshold, corrected for multiple testing.

To verify that our random permutation test works correctly, all the preprocessing steps were removed and Gaussian white noise was used as data. The stimulus paradigm convolved with the HRF and its temporal derivative were used as regressors, and a *t*-test value was calculated for each voxel. A spatial mask from a real fMRI dataset was used to get the same number of brain voxels. A threshold for corrected *P* = 0.05 was calculated, by using 100 000 permutations, and then 10 000 noise datasets were generated (for each amount of smoothing), analysed, and thresholded. If the calculated threshold is correct, 500 of the noise datasets should contain a test value that is higher than the threshold. The family-wise error rate (FWE) was estimated for the thresholds from Bonferroni correction, Gaussian random field theory, and the random permutation test and is given in Figure 7.

### 4.5. GLM versus CCA

**Figure 8:**
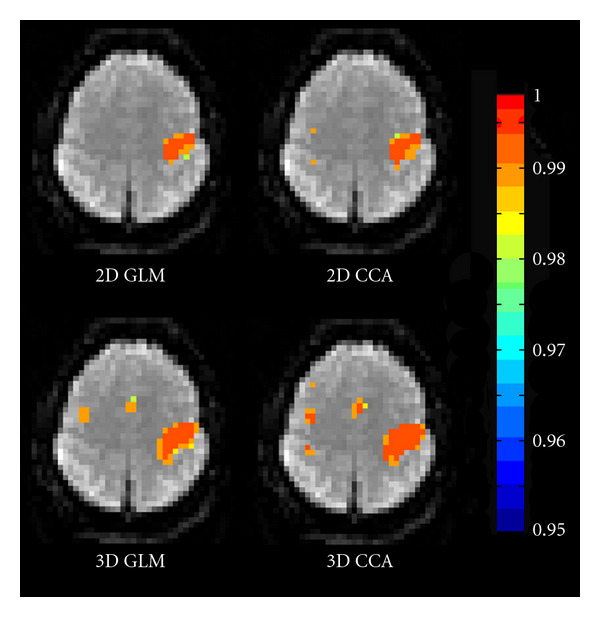
**Top**: A comparison between corrected *P* values from 2D GLM (left) and 2D CCA (right), calculated from a random permutation test with 10 000 permutations. The activity maps are thresholded at the same significance level (corrected *P* = 0.05). The GLM used one isotropic 8 mm FWHM 2D Gaussian smoothing kernel while CCA used one isotropic 2D Gaussian kernel and 3 anisotropic 2D Gaussian kernels, designed such that the largest possible filter that CCA can create has an FWHM of 8 mm. The neurological display convention is used (left is left), 1−*p* is shown instead of *p*. Note that CCA detects active voxels in the left motor cortex and in the left somatosensory cortex that are not detected by the GLM. **Bottom**: A comparison between corrected *P* values from 3D GLM (left) and 3D CCA (right), calculated from a random permutation test with 10 000 permutations. The activity maps are thresholded at the same significance level (corrected *P* = 0.05). The GLM used one isotropic 8 mm FWHM 3D Gaussian smoothing kernel while CCA used one isotropic 3D Gaussian kernel and its derivative, designed such that the largest possible filter that CCA can create has a FWHM of 8 mm. The neurological display convention is used (left is left), 1−*p* is shown instead of *p*. Note that CCA detects active voxels in the left somatosensory cortex that are not detected by the GLM.

With the random permutation test it is possible to calculate corrected *P* values for fMRI analysis by CCA, and thereby activity maps from GLM and CCA can finally be compared at the same significance level. The activity maps are given in Figure 8. For these comparisons, the *Motor 1* dataset was used, 10 000 permutations were used both for GLM and CCA. The activity maps were thresholded at the same significance level, corrected *P* = 0.05. With 8 mm of 2D smoothing, GLM detects 302 significantly active voxels while CCA detects 344 significantly active voxels. With 8 mm of 3D smoothing, GLM detects 475 significantly active voxels while CCA detects 684 significantly active voxels. The aim of this comparison is not to prove that CCA has a superior detection performance, but to show that objective evaluation of different methods for single-subject fMRI analysis becomes practically possible by using fast random permutation tests. 

**Figure 9:**
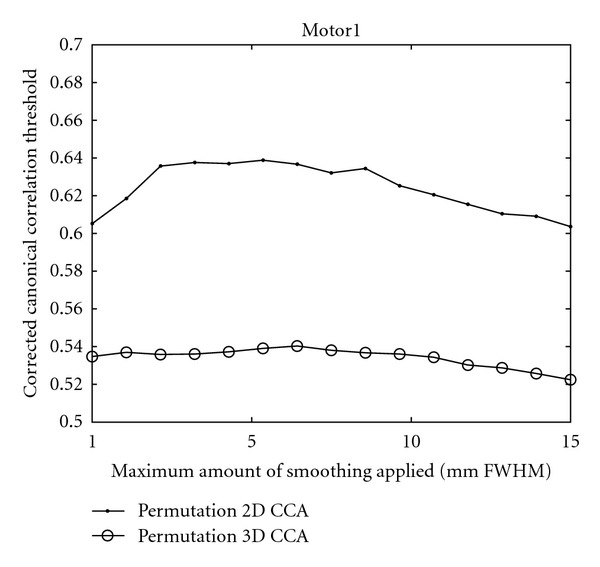
Canonical correlation thresholds, for corrected *P* = 0.05, as function of the maximum amount of smoothing that can be applied by CCA. The Motor 1 dataset was used.

For RCCA there is no theoretical distribution to calculate a threshold from, and therefore the corrected thresholds for the restricted canonical correlation coefficients are also presented, 10 000 permutations were used to calculate each threshold.  Figure 9 shows the found thresholds for 2D CCA and 3D CCA for the *Motor 1* dataset. Similar results were obtained for the other datasets. Since fMRI analysis by CCA results in an adaptive smoothing, compared to a fix smoothing with the GLM, the amount of smoothing varies between the voxels. Due to this, the plots show the corrected thresholds for the different *maximum* amounts of smoothing that can be applied by CCA. These plots would have taken a total of about 14 days to generate with a standard *C* implementation, with our multi-GPU implementation they took about 30 minutes to generate.

### 4.6. Comparison of Methods for Calculating Corrected Thresholds

As the null distribution of the maximum *t*-test statistics can be estimated, it is possible to compare the thresholds that are given by Bonferroni correction, Gaussian random field theory, and a random permutation test (which should give the most correct threshold); 10 000 permutations were used for the random permutation test.

**Figure 10:**
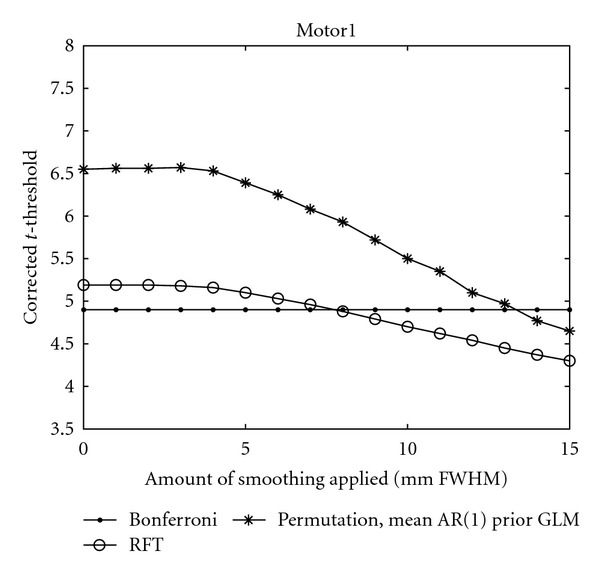
A comparison of *t*-thresholds, for corrected *P* = 0.05, from three approaches to calculate a corrected threshold, as function of the amount of smoothing applied. The Motor 1 dataset was used.

Figure 10 shows the found thresholds for the *Motor 1* dataset, for different amounts of smoothing. Similar results were obtained for the other datasets. To our knowledge, a comparison of thresholds from Bonferroni correction, Gaussian random field theory, and a random permutation test has previously only been done for *multi-subject* fMRI [24]. These plots would have taken a total of about 5.5 days to generate with a standard *C* implementation; with our multi-GPU implementation they took about 38 minutes to generate.

**Figure 11:**
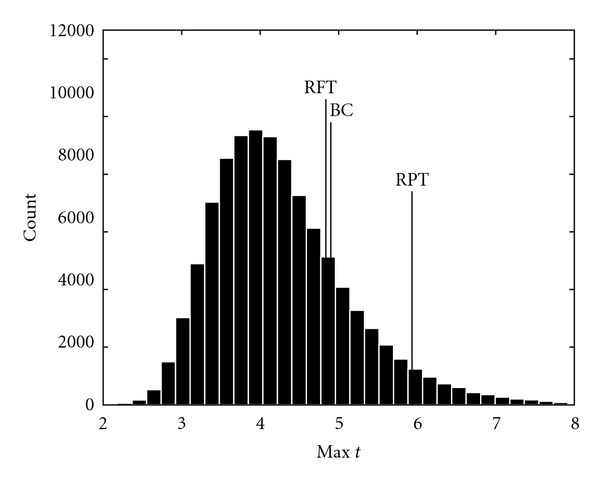
The estimated null distribution of the maximum *t*-test value from the GLM, when 8 mm smoothing was applied. The calculated thresholds for corrected *P* = 0.05 for the three approaches are marked with BC (Bonferroni correction), RFT (random field theory), and RPT (random permutation test).

Figure 11 shows the estimated maximum *t* distribution for the *Motor 1* dataset; 8 mm of smoothing was applied to the volumes in each permutation. 

### 4.7. Distributions of Corrected *t*-Thresholds

**Figure 12:**
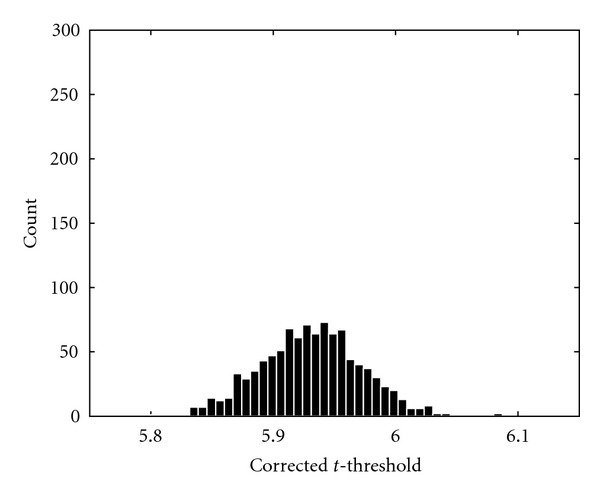
The distribution of the corrected *t*-threshold when 1000 permutations were used.

**Figure 13:**
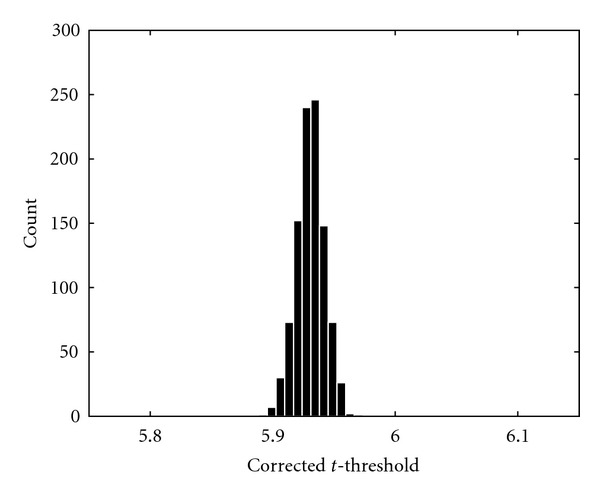
The distribution of the corrected *t*-threshold when 10 000 permutations were used.

As a final result, distributions of the corrected *t*-thresholds are presented. The random permutation test for the GLM was repeated 1000 times and the resulting thresholds were saved. The *Motor 1* dataset was used with 8 mm of smoothing. The threshold distribution for 1000 permutations is given in Figure 12 and the threshold distribution for 10 000 permutations is given in Figure 13. The standard deviation of the threshold calculated with 1000 permutations is 0.0364, and the standard deviation of the threshold calculated with 10 000 permutations is 0.0114. According to [53] the standard deviation should decrease with $\sqrt{10}$ if 10 times more permutations are used. The difference in standard deviation between the threshold calculated with 1000 and 10 000 permutations is very close to this approximation.

If 1000 permutations are used (and it is assumed that the estimated distribution is correct), the estimated *P* value varies between 0.044 and 0.059 if the standard deviation of the corrected threshold is subtracted or added. For 10 000 permutations the estimated *P* value varies between 0.048 and 0.052. This is close to the expected relative standard deviation given in Table 1. It is very important to know the variance of the *P* values as it tells us how reliable the estimates are.

These plots would have taken a total of about 17.4 and 174 days to generate with a standard *C* implementation. With the multi-GPU implementation they took about 2.3 and 18 hours to generate.

## 5. Discussion

 With the help of fast random permutation tests it is possible to objectively evaluate activity maps from any test statistics easily, by using the same significance level. As an example of this we compare activity maps from GLM and CCA. It is also possible to investigate how a change in the preprocessing (e.g., the amount of smoothing or the whitening applied) affects the distribution of the maximum test statistics. The search for the best test statistics, that gives the best separation between active and inactive voxels, can now be started. To use simple test statistics and hope that the data is normally distributed and independent is no longer necessary.

### 5.1. Processing Times

As can be seen in the tables, a lot of time is saved by using the GPU. Most of the time is saved in the smoothing step. The tables clearly show that the GPU, or an advanced PC cluster, is a must for random permutation tests that include smoothing. To do 100 000 permutations with CCA takes about 7 days with the *C* implementation, about a day with the OpenMP implementation, and about 9 minutes with the multi-GPU implementation. The speedup is about 1100 between the *C* implementation and the multi-GPU implementation and about 170 between the OpenMP implementation and the multi-GPU implementation.

It should be noted that these processing times are for 80 volumes and 20 000 brain voxels, but it is not uncommon that an fMRI dataset contains 150 volumes and 30 000 brain voxels, which triples the processing times.

The main problem with a long processing time is the software development. In order to test and verify that a program works correctly, the program has to be launched a large number of times. During the development of the routines and the writing of the paper we ran the complete analysis, with 1000–100 000 permutations, at least 3000 times. For the GLM this means that at least 6000 hours of processing time were saved, compared to the *C* implementation. This is equivalent to 750 working days.

With the power of the GPU it is even possible to look at the distributions of the corrected thresholds that otherwise could take as much as 6 months of processing time to estimate.

The processing time for 10 000 permutations with GLM and smoothing is about 3.5 minutes with one GPU. This is perhaps too long for clinical applications, but we believe that it is fast enough for researchers to use it in their daily work.

### 5.2. GLM versus CCA

With the help of the GPU it is finally possible to compare activity maps from GLM and CCA at the same significance level. Even if CCA has a superior detection performance compared to the GLM, its use has been limited. One major reason for this is that it is hard to set a (corrected) threshold for CCA.

The presented activity maps show that the CCA approach in general, due to its spatial adaptivity, finds a higher number of significantly active voxels than the GLM approach. With 2D smoothing CCA finds some significantly active voxels in the left motor cortex and in the left somatosensory cortex that are not detected by the GLM. With 3D smoothing CCA finds some significantly active voxels in the left somatosensory cortex that are not detected by the GLM. We have thereby confirmed previous results that fMRI analysis by CCA can result in a higher number of significantly active voxels [9, 10, 56].

It might seem strange that the corrected canonical correlation thresholds do not decrease as rapidly as the corrected *t*-thresholds when the maximum amount of smoothing increases. By using CCA an adaptive smoothing is obtained, such that the amount of smoothing varies between the voxels. The CCA approach will choose the amount (and orientation) of smoothing that results in the highest canonical correlation, as shown in Figure 3. This is one of the main advantages with CCA, since it, for example, prevents that too much smoothing is used in small activity areas. If the maximum canonical correlation is found by only using the small lowpass filter, the maximum canonical correlation might not change significantly when the maximum amount of smoothing is increased since CCA probably will choose to only use the small lowpass filter once again. The consequence is that it is hard to predict how the maximum test value will change as a function of the maximum amount of smoothing.

The corrected thresholds are lower for 3D CCA than for 2D CCA. This is explained by the fact that the 2D version is adaptive in both scale and orientation, and it can thereby find higher correlations than the 3D version that only is adaptive in scale. With more advanced GPUs, the original 3D CCA approach, with 7 filters, can be used to obtain more spatial adaptivity in 3D.

### 5.3. Comparison of Methods for Calculating Corrected Thresholds

The comparison between the thresholds from Bonferroni correction, Gaussian random field theory, and the random permutation test shows some interesting results. The thresholds from the random permutation test are the highest. For the GLM approach to be valid, the data is assumed to be normally distributed as well as independent. For the multiple testing problem, the parametric approaches also assume a common null distribution for each voxel while the permutation approach does not [24]. There are thus, at least, three sources of error for the parametric approaches.

As a *t*-value is calculated for each time series, the normality condition should be investigated for each time series separately [2], for example, by a Kolmogorov-Smirnov test or a Shapiro-Wilk test. These tests are, however, not very reliable if there are only 80 time points for each voxel. The *maximum t*-distribution is very sensitive to deviations from normality [57], while the standard *t*-distribution is rather robust. If a few voxel time series have a distribution that deviates from normality, this is sufficient to affect the maximum *t*-distribution and thereby the threshold [24]. This will be captured by the random permutation test but not by the parametric tests.

The distribution of the *t*-test values from the *Null* dataset does not strictly follow a Student's *t*-distribution, especially if 10 mm smoothing is used. The tails are not longer but slightly thicker than a true *t*-distribution. When a mean AR(1) whitening was used for a conventional analysis of the *Null* dataset, the *t*-test value that is bigger than 95% of the test values is 1.75 when no smoothing is used, 1.66 when 5 mm of smoothing is used, and 1.59 when 10 mm smoothing is used. The theoretical threshold for uncorrected *P* = 0.05, calculated from the Student's *t*-distribution, is 1.66. This explains why the thresholds from the random permutation test are higher than the thresholds from Bonferroni correction.

It is commonly assumed that the noise in MRI is normally distributed, but due to the fact that only the magnitude of the MRI data is used, the noise is actually Rician distributed [1]. The original (complex valued) noise in MRI is normally distributed, but the magnitude operation after the inverse Fourier transform in the image reconstruction process is not linear and thereby changes the distribution of the noise. The distribution of the fMRI noise is more complicated as there are several sources of artefacts and the difference between images is used to calculate the test statistics [2–5]. The consequence for fMRI is that the residuals from the GLM might not be normally distributed, even if the model is valid. For the model to be valid, all possible artefacts that can arise have to be modelled. This includes motion-related artefacts, breathing artefacts, pulse artefacts, and MR scanner imperfections. To make a perfect model for all these artefacts is a big challenge on its own.

Another problem for the random field theory approach is that the activity map has to be sufficiently smooth in order to approximate the behaviour of a continuous random field. The smoothness also has to be estimated from the data and it is assumed that it is constant in the brain. These assumptions and several others [24, 39] have to be met in order for the random field theory approach to be valid. For the random permutation test some assumptions also have to be made, for example that the time series are correctly whitened before the permutations. The number of necessary assumptions for the random permutation test is, however, significantly lower than that for the parametric approaches.

### 5.4. Future Work

In this paper we have only described what is known as a *single-threshold* permutation test, but other types of permutation tests can be more powerful. Examples of this are the so-called *step-down* and *step-up* permutation tests. These permutation tests are even more computationally demanding; it can, for example, be necessary to reestimate the maximum null distribution for each voxel. It is also possible to use the mass of a cluster [28, 58] instead of the voxel intensity, or a combination of the voxel intensity and the cluster extent [25]. The random permutation tests can also be used in order to calculate significance thresholds for functional connectivity analysis [31, 32].

The GPU can of course also be used to speed up permutation tests for multi-subject fMRI and multi-subject PET, and not only for single-subject fMRI. The only drawback with the GPU that has been encountered so far is that some test statistics, like 3D CCA, are harder to implement on the GPU than on the CPU, due to the current limitations of the GPU. It must also be possible to calculate the test statistics in parallel, otherwise the GPU will not provide any speedup.

## 6. Conclusions

We have presented how to apply random permutation tests for single-subject analysis of fMRI data by using the graphics processing unit (GPU). Our work enables objective evaluation of arbitrary methods for single-subject fMRI analysis. As a pleasant side effect, the problem of multiple testing is solved in a way that significantly reduces the number of necessary assumptions. To our knowledge, our implementation is the first where the smoothing is done *in each permutation*. In previous papers about permutation tests in fMRI, it is neglected that the smoothing has to be done in each permutation for the analysis to be correct.

## Figures and Tables

**Algorithm 1:**
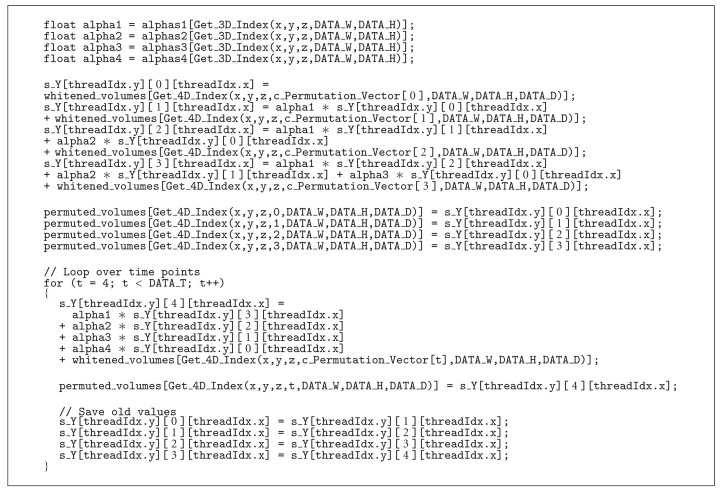


**Table 1:** Relative standard deviation of the desired *P* value, as function of desired *P* value and number of permutations.

Number of Permutations/*P* value	0.1	0.05	0.01
1000	9.48%	13.78%	31.46%
5 000	4.24%	6.16%	14.07%
10 000	3%	4.35%	9.95%
50 000	1.34%	1.94%	4.45%
100 000	0.95%	1.37%	3.14%

**Table 2:** Processing times for random permutation tests with the GLM for the different implementations.

Number of permutations with GLM	*C *	OpenMP	CUDA, 1 × GTX 480	CUDA, 3 × GTX 480
1000	25 min	3.5 min	25.2 s	8.4 s
5 000	2 h 5 min	17.5 min	1 min 42 s	33.9 s
10 000	4 h 10 min	35 min	3 min 18 s	65.8 s
50 000	20 h 50 min	2 h 55 min	16 min 30 s	5 min 30 s
100 000	1 day 17 h 40 min	5 h 50 min	33 min	11 min

**Table 3:** Processing times for random permutation tests with 2D CCA for the different implementations.

Number of permutations with 2D CCA	C	OpenMP	CUDA, 1 × GTX 480	CUDA, 3 × GTX 480
1000	1 h 40 min	14 min 50 s	22.2 s	7.4 s
5 000	8 h 20 min	1 h 14 min	1 min 24 s	28 s
10 000	16 h 37 min	2 h 28 min	2 min 42 s	54 s
50 000	3 days 11 h	12 h 22 min	13 min 30 s	4 min 30 s
100 000	6 days 22 h	24 h 43 min	27 min	9 min

## References

[1] Gudbjartsson H, Patz S (1995). The Rician distribution of noisy MRI data. *Magnetic Resonance in Medicine*.

[2] Luo WL, Nichols TE (2003). Diagnosis and exploration of massively univariate neuroimaging models. *NeuroImage*.

[3] Friman O, Morocz I, Westin C-F Examining the whiteness of fMRI
noise.

[4] Lund TE, Madsen KH, Sidaros K, Luo WL, Nichols TE (2006). Non-white noise in fMRI: does modelling have an impact?. *NeuroImage*.

[5] Wink AM, Roerdink JBTM (2006). BOLD noise assumptions in fMRI. *International Journal of Biomedical Imaging*.

[6] Friston KJ, Josephs O, Zarahn E, Holmes AP, Rouquette S, Poline JB (2000). To smooth or not to smooth? Bias and efficiency in fMRI time-series analysis. *NeuroImage*.

[7] Worsley KJ, Liao CH, Aston J (2002). A general statistical analysis for fMRI data. *NeuroImage*.

[8] Friman O, Cedefamn J, Lundberg P, Borga M, Knutsson H (2001). Detection of neural activity in functional MRI using canonical correlation analysis. *Magnetic Resonance in Medicine*.

[9] Friman O, Borga M, Lundberg P, Knutsson H (2003). Adaptive analysis of fMRI data. *NeuroImage*.

[10] Nandy R, Cordes D (2003). A novel nonparametric approach to canonical correlation
analysis with applications to low CNR functional MRI data. *Magnetic Resonance in Medicine*.

[11] Mourão-Miranda J, Bokde ALW, Born C, Hampel H, Stetter M (2005). Classifying brain states and determining the discriminating activation patterns: support Vector Machine on functional MRI data. *NeuroImage*.

[12] Kriegeskorte N, Goebel R, Bandettini P (2006). Information-based functional brain mapping. *Proceedings of the National Academy of Sciences of the United States of America*.

[13] Martino FD, Valente G, Staeren N, Ashburner J, Goebel R, Formisano E (2008). Combining multivariate voxel selection and support vector machines for mapping and classification of fMRI spatial patterns. *NeuroImage*.

[14] Åberg MB, Wessberg J (2008). An evolutionary approach to the identification of informative voxel clusters for brain state discrimination. *IEEE Journal on Selected Topics in Signal Processing*.

[15] Hochberg Y, Tamhane AC (1987). *Multiple Comparison Procedures*.

[16] Siegel S (1957). Nonparametric statistics. *The American Statistician*.

[17] Holmes AP, Blair RC, Watson JDG, Ford I (1996). Nonparametric analysis of statistic images from functional mapping experiments. *Journal of Cerebral Blood Flow & Metabolism*.

[18] Bullmore E, Brammer M, Williams SCR (1996). Statistical methods of estimation and inference for functional MR image analysis. *Magnetic Resonance in Medicine*.

[19] Locascio JJ, Jennings PJ, Moore CI, Corkin S (1997). Time series analysis in the time domain and resampling methods for studies of functional magnetic resonance brain imaging. *Human Brain Mapping*.

[20] Brammer MJ, Bullmore ET, Simmons A (1997). Generic brain activation mapping in functional magnetic resonance imaging: a nonparametric approach. *Magnetic Resonance Imaging*.

[21] Belmonte M, Yurgelun-Todd D (2001). Permutation testing made practical for functional magnetic resonance image analysis. *IEEE Transactions on Medical Imaging*.

[22] Bullmore E, Long C, Suckling J (2001). Colored noise and computational inference in neurophysiological (fMRI) time series analysis: resampling methods in time and wavelet domains. *Human Brain Mapping*.

[23] Nichols TE, Holmes AP (2002). Nonparametric permutation tests for functional neuroimaging: a primer with examples. *Human Brain Mapping*.

[24] Nichols T, Hayasaka S (2003). Controlling the familywise error rate in functional neuroimaging: a comparative review. *Statistical Methods in Medical Research*.

[25] Hayasaka S, Nichols TE (2004). Combining voxel intensity and cluster extent with permutation test framework. *NeuroImage*.

[26] Breakspear M, Brammer MJ, Bullmore ET, Das P, Williams LM (2004). Spatiotemporal wavelet resampling for functional neuroimaging data. *Human Brain Mapping*.

[27] Friman O, Westin CF (2005). Resampling fMRI time series. *NeuroImage*.

[28] Tillikainen L, Salli E, Korvenoja A, Aronen HJ (2006). A cluster mass permutation test with contextual enhancement for fMRI activation detection. *NeuroImage*.

[29] Nandy R, Cordes D (2007). A semi-parametric approach to estimate the family-wise error rate in fMRI using resting-state data. *NeuroImage*.

[30] Eklund A, Andersson M, Knutsson H fMRI analysis on the GPU-possibilities and challenges.

[31] Gembris D, Neeb M, Gipp M, Kugel A, Männer R (2010). Correlation analysis on GPU systems using NVIDIA’s CUDA. *Journal of Real-Time Image Processing*.

[32] Eklund A, Friman O, Andersson M, Knutsson H A GPU accelerated interactive interface for exploratory functional connectivity analysis of fMRI data.

[33] Ferreira da Silva AR (2011). A bayesian multilevel model for fMRI data analysis. *Computer Methods and Programs in Biomedicine*.

[34] Shterev I, Jung S-H, George S, Owzar K (2010). permGPU: using graphics
processing units in RNA microarray association studies. *BMC Bioinformatics*.

[35] Hemert JLV, Dickerson JA (2011). Monte Carlo randomization tests for large-scale abundance datasets on the GPU. *Computer Methods and Programs in Biomedicine*.

[36] Friston KJ, Jezzard P, Turner R (1993). Analysis of functional MRI time-series. *Human Brain Mapping*.

[37] Friston KJ, Holmes AP, Worsley KJ, Poline JP, Frith CD, Frackowiak RSJ (1994). Statistical parametric maps in functional imaging: a general linear approach. *Human Brain Mapping*.

[38] Kiebel SJ, Poline JB, Friston KJ, Holmes AP, Worsley KJ (1999). Robust smoothness estimation in statistical parametric maps using standardized residuals from the general linear model. *NeuroImage*.

[39] Frackowiak RS, Friston K, Frith C (2004). *Human Brain Function*.

[40] Dwass M (1957). Modified randomization tests for nonparametric hypotheses. *The Annals of Mathematical Statistics*.

[41] Smith AM, Lewis BK, Ruttimann UE (1999). Investigation of low frequency drift in fMRI signal. *NeuroImage*.

[42] Friman O, Borga M, Lundberg P, Knutsson H (2004). Detection and detrending in fMRI data analysis. *NeuroImage*.

[43] Laird AR, Rogers BP, Meyerand ME (2004). Comparison of Fourier and wavelet resampling methods. *Magnetic Resonance in Medicine*.

[44] Gautama T, Van Hulle MM (2004). Optimal spatial regularisation of autocorrelation estimates in fMRI analysis. *NeuroImage*.

[45] Lenoski B, Baxter LC, Karam LJ, Maisog J, Debbins J (2008). On the performance of autocorrelation estimation algorithms for fMRI analysis. *IEEE Journal on Selected Topics in Signal Processing*.

[46] Knutsson H, Westin C-F Normalized and differential convolution: methods for interpolation and filtering of incomplete and uncertain data.

[47] Ljung GM, Box GEP (1978). On a measure of lack of fit in time series models. *Biometrika*.

[48] Hotelling H (1936). Relation between two sets of variates. *Biometrika*.

[49] Nguyen TK, Eklund A, Ohlsson H Concurrent volume visualization
of real-time fMRI.

[50] Constantine A (1963). Some non-central distribution problems in multivariate
analysis. *Annals of Mathematical Statistics*.

[51] Das S, Sen PK (1994). Restricted canonical correlations. *Linear Algebra and Its Applications*.

[52] Friman O Subspace models for functional MRI data analysis.

[53] Moore DS, McCabe GP, Craig BA (2007). *Introduction to the Practice of Statistics*.

[55] Kirk D, Hwu W (2010). *Programming Massively Parallel Processors, A Hands on Approach*.

[56] Ragnehed M, Engström M, Knutsson H, Söderfeldt B, Lundberg P (2009). Restricted canonical correlation analysis in functional MRI-validation and a novel thresholding technique. *Journal of Magnetic Resonance Imaging*.

[57] Viviani R, Beschoner P, Ehrhard K, Schmitz B, Thöne J (2007). Non-normality and transformations of random fields, with an application to voxel-based morphometry. *NeuroImage*.

[58] Bullmore ET, Suckling J, Overmeyer S, Rabe-Hesketh S, Taylor E, Brammer MJ (1999). Global, voxel, and cluster tests, by theory and permutation, for a difference between two groups of structural MR images of the brain. *IEEE Transactions on Medical Imaging*.

